# An Improved Linear Spectral Emissivity Constraint Method for Temperature and Emissivity Separation Using Hyperspectral Thermal Infrared Data

**DOI:** 10.3390/s19245552

**Published:** 2019-12-16

**Authors:** Xinyu Lan, Enyu Zhao, Zhao-Liang Li, Jélila Labed, Françoise Nerry

**Affiliations:** 1 ICube, UdS, CNRS, 300 Bld Sébastien Brant, CS10413, 67412 Illkirch, France; xinyu.lan@etu.unistra.fr (X.L.); lizl@unistra.fr (Z.-L.L.); labed@unistra.fr (J.L.); f.nerry@unistra.fr (F.N.); 2 Information Science and Technology College, Dalian Maritime University, Dalian 116026, China

**Keywords:** linear spectral emissivity constraint, land surface temperature (LST), land surface emissivity (LSE), hyperspectral thermal infrared

## Abstract

The linear spectral emissivity constraint (LSEC) method has been proposed to separate temperature and emissivity in hyperspectral thermal infrared data with an assumption that land surface emissivity (LSE) can be described by an equal interval piecewise linear function. This paper combines a pre-estimate shape method with the LSEC method to provide an initial-shape estimation of LSE which will create a new piecewise scheme for land surface temperature (LST) and LSE separation. This new scheme is designated as the pre-estimate shape (PES)-LSEC method. Comparisons with the LSEC method using simulated data sets show that the PES-LSEC method has better performance in terms of accuracy for both LSE and LST. With an at-ground error of 0.5 K, the root-mean-square errors (RMSEs) of LST and LSE are 0.07 K and 0.0045, respectively, and with the scale factor of moisture profile 0.8 and 1.2, the RMSEs of LST are 1.11 K and 1.14 K, respectively. The RMSEs of LSE in each channel are mostly below 0.02 and 0.04, respectively, which are better than for the LSEC method. In situ experimental data are adopted to validate our method: The results show that RMSE of LST is 0.9 K and the mean value of LSE accuracy is 0.01. The PES-LSEC method with fewer segments achieves better accuracy than that of LSEC and preserves most of the crest and trough information of emissivity.

## 1. Introduction

Hyperspectral thermal infrared data (TIR) with more refined spectral characteristics provide a great deal of information on land surface processes, especially land surface temperature (LST) [1]. Currently, hyperspectral TIR data can be obtained from hyperspectral infrared sensors, such as the Atmospheric InfraRed Sounder (AIRS), the Infrared Atmospheric Sounding Interferometer (IASI), and the Cross-track Infrared Sounder (CrIS). However, in the thermal infrared region, accurately retrieving LST, which is tightly coupled with land surface emissivity (LSE), is an ill-posed problem because the number of unknowns (1 temperature and N emissivities) is larger than the number of equations (N spectral bands), even if an accurate atmospheric correction has been achieved. On the basis of this problem, many methods have been proposed to solve the underdetermined equations to separate the LST and LSE to obtain accurate solutions using hyperspectral TIR data.

Some indoor or ground-based experiments made great efforts to separate the surface temperature and emissivity for hyperspectral data. For the ‘Planck draping’ method [2], the retrieval temperature is that which gives the best fit of the Planck’s function to the measured spectra, with the assumption that the maximum emissivity is 0.97. The authors in [3,4] presented a method in which the sample temperature is varied to minimize the residual atmospheric emission lines in the measured field emissivity spectra. With the control of temperature using the heating source, the downwelling radiance is a critical parameter in calculating the emissivity. The non-negative matrix factorization provides an unsupervised linear representation of the data similar to principal component analysis (PCA) by using non-negative coefficients in the calculation of eigenvalues, thus it is widely adopted to determine the downwelling radiance and further calculate the emissivity [5,6,7].

Indeed, without an accurate atmospheric correction, the artificial neural network (ANN) method [8,9], physical method [10,11,12,13], and multi-channel method [14] are commonly used to provide us with LST information for the satellite level. With accurate atmospheric correction, there are mainly two ways to solve the problem. The first way is based on the spectral characteristics of physical quantities. The iterative spectrally smooth temperature and emissivity separation (ISSTES) algorithm is commonly used based on the assumption that the emissivity spectrum is smoother than the atmospheric spectrum in hyperspectral TIR data [15]. With many studies and improvements, this method currently presents satisfactory performances [16,17]. Meanwhile, the correlation-based method [18], downwelling radiance residual Index (DRRI) [19], and stepwise refining algorithm [20] can also be used to obtain better solutions for calculating the LST and LSE without considering the atmospheric error influence. The second approach is via the descending dimension of N channels emissivity, thus reducing the number of the unknowns. Linear spectral emissivity constraint (LSEC) [21] and wavelet transform [22] successfully turn the underdetermined problem into an overdetermined problem, making the temperature-emissivity separation solvable. Compared with ISSTES method, LSEC method is efficient and easily implemented because there are no singular points in its cost function. If the data include noise, the LSEC method also can produce more accurate results than the ISSTES method with good noise-resistant ability [21]. However, using an equal piecewise linear LSE description (segment length 10 cm^−1^) will result in some crests or troughs of emissivity being directly covered by straight lines, thus losing their information. If we can get the real or an estimated shape of emissivity and propose a new segmentation scheme according to its shape, the accuracy or the operational efficiency will be improved to some extent.

In this paper, an initial-shape-estimation algorithm of LSE is proposed to provide a new LSE segmentation scheme that is subsequently adopted in the separation of LST and LSE for simulated hyperspectral infrared IASI data. Section 2 is devoted to the methodological development, describing the theoretical basis and improvement of retrieving LST. Section 3 gives the simulated numerical experiment and sensitivity analysis. Section 4 presents the validation with in situ measurements. Finally, the conclusions are summarized in the last section.

## 2. Methodology

Assuming a cloud-free atmosphere under local thermodynamic equilibrium and neglecting the atmospheric scattering effects, in the TIR region, the radiative transfer equation (RTE) can be written as [23]

(1) $$L\left(\lambda ,\theta ,\varphi \right)=\varepsilon \left(\lambda ,\theta ,\varphi \right)B\left({\lambda ,T}_{s}\right)\tau \left({\lambda ,\theta ,\varphi ,p}_{s}\right)+\int_{P_{s}}^{0}B\left({\lambda ,T}_{p}\right)\frac{\partial \tau \left(\lambda ,\theta ,\varphi ,p\right)}{\partial p}\mathrm{dp}\;+\int_{0}^{2\pi }\int_{0}^{\frac{\pi }{2}}\rho \left(\lambda ,\theta ,\varphi ,\;\theta^{\prime }\;,\;\varphi^{\prime }\right)L_{d}\left(\lambda ,\;\theta^{\prime }\;,\;\varphi^{\prime }\right)\tau \left({\lambda ,\theta ,\varphi ,p}_{s}\right)\cos\;\theta^{\prime }\;\sin\;\theta^{\prime }\;d\;\theta^{\prime }\;d\;\varphi^{\prime }$$

where λ is wavelength; θ and φ are the viewing zenith angle and azimuth angle, respectively; $\theta^{\prime }$ and $\varphi^{\prime }$ are the zenith angle and azimuth angle of downwelling direction of atmospheric radiance, respectively; L is the measured spectral radiance at the top of the atmosphere (TOA); ε is the LSE; B (λ,T_s_) is the Planck function at surface temperature T_s_; P_s_ is the surface pressure level; τ is the atmospheric transmittance from a pressure level to the TOA along the viewing angle; T_p_ is atmospheric temperature; and ρ is surface bidirectional reflectance. Assuming the surface as a Lambertian reflector, ρ= (1−ε)/π is also substituted into Equation (1). $\frac{1}{\pi }\int_{0}^{2\pi }\int_{0}^{\frac{\pi }{2}}L_{d}\left(\lambda ,\;\theta^{\prime }\;,\;\varphi^{\prime }\right)\cos\;\theta^{\prime }\;\sin\;\theta^{\prime }\;d\;\theta^{\prime }\;d\;\varphi^{\prime }$ is atmospheric downwelling radiance, it is written as R_down_ (λ). $\tau \;\left({\lambda ,\theta ,\varphi ,p}_{s}\right)$ is written as τ(λ). L(λ,θ,φ) is expressed as L_m_ (λ). $\int_{P_{s}}^{0}B\left({\lambda ,T}_{p}\right)\frac{\partial \tau \left(\lambda ,\theta ,\varphi ,p\right)}{\partial p}\mathrm{dp}$ is the atmospheric upwelling radiance, and is written as R_up_ (λ), then we obtain Equation (2).

L_m_(λ) = [ε(λ)B(λ,T_s_) + (1-ε(λ))R_down_(λ)]τ(λ) + R_up_(λ). (2)

All elements in Equation (2), excluding the LST, which is regarded as constant in all bands (wavelength), are wavelength-dependent for hyperspectral sensors (N bands). 

**Figure 1:**
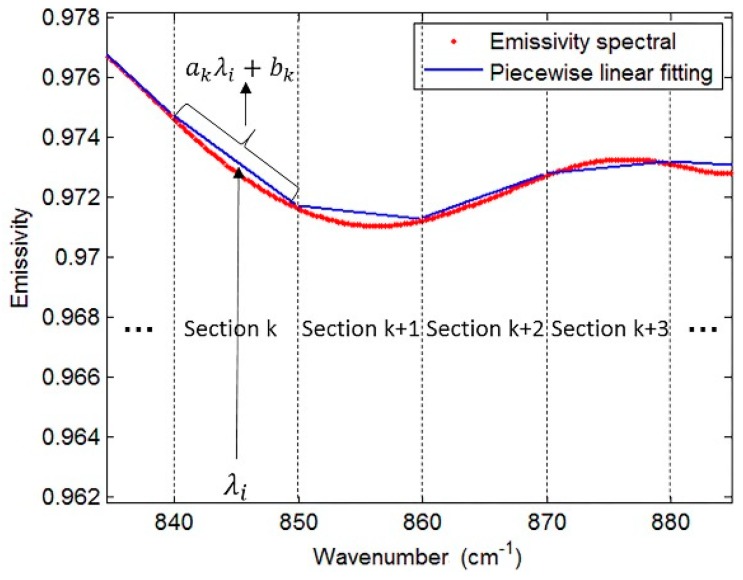
Diagrammatic sketch of piecewise linear emissivity spectra fitting. The red line is an actual emissivity spectrum (from a type of soil), the abscissa is the wavenumber, and the ordinate is the emissivity, while the blue lines are the fitting spectra.

When an accurate atmospheric correction has been done (τ, R_up_, and R_down_ for all bands are known), N equations will contain N unknown emissivities plus one unknown temperature, making the system of equations underdetermined. Many approaches have been developed to overcome the problem of having an underdetermined system of equations. Compared to other published methods, LSEC is simpler and more efficient, with a strong anti-noise ability. In order to make equations solvable and further retrieve LST in LSEC, LSE is approximated as a piecewise linear function (M sections). As shown in Figure 1, LSE is divided into M sections and every section can be expressed using a linear function, thus the *ith* channel’s emissivity within the *kth* section can be expressed as a linear function with channel (wavelength) λ_i_ (Equation (3)).

ε_k_ (λ_i_) ≈ a_k_λ_i_ + b_k_, k=1,…,M, (3)

where a_k_ and b_k_ are the two sets of coefficients of the M linear functions.

**Figure 2:**
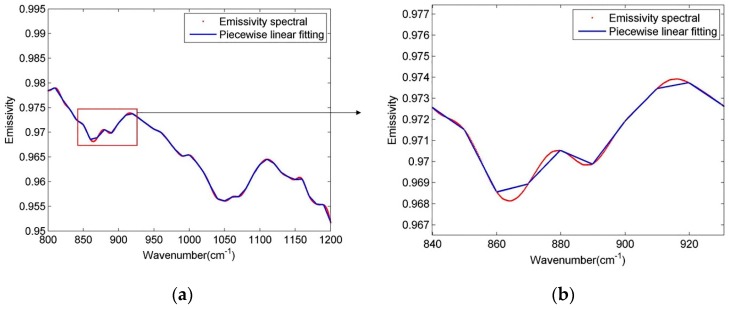
Diagrammatic sketch of piecewise linear emissivity spectra fitting. Figure (**b**) is a partial enlarged view of figure (**a**). The red line is an actual emissivity spectrum (from a type of soil), while the blue lines are the fitting spectra.

This linear approximation of the segment reduces the number of unknowns, making the system of equations solvable. Meanwhile, accurate atmospheric correction is necessary to avoid significantly deteriorated and unacceptable accuracy of LST. However, the equal interval scheme, with an equal interval segment length of 10 cm^−1^ as suggested in the LSEC method, causes some feature points (crest or trough points of the spectrum) to be lost. Figure 2 shows that with a 10 cm^−1^ segment length, some crests and troughs on the curve are covered, which means the scheme does not satisfactorily fit the real spectrum curve. Moreover, for a spectrum that resembles a straight line in a wider band, segmentation with 10 cm^−1^ leads to too many segments, which will affect the calculation efficiency. 

If the length of segment can be changed according to the variation of the actual emissivity spectrum, the fitting may be further improved in terms of accuracy and speed of operation. Therefore, this work provides a procedure for estimating the initial shape of LSE to keep most of the crest and trough information, then discusses whether the initial-shape-estimation process will lead to better accuracy. After compensating for the atmosphere (downwelling radiance, upwelling radiance, and transmittance are known), we attempt to estimate the shape of the unknown LSE. L_sur_ (λ) is adopted to express (L_m_(λ)−R_up_(λ))/τ(λ), then Equation (4) is another form of Equation (2): LSE can be calculated with an initial estimation of LST (T_s_).

ε(λ) = (L_sur_(λ) − R_down_(λ))/(B(λ,T_s_) − R_down_(λ)). (4)

The deviation of LSE caused by the error of estimated LST implies the influence of atmospheric downwelling radiation. That is, when the temperature estimation is biased, the obtained emissivity spectrum will have the spectral characteristics of atmospheric downwelling radiation (absorption line characteristics, showing more twists and turns, not smooth). 

**Figure 3:**
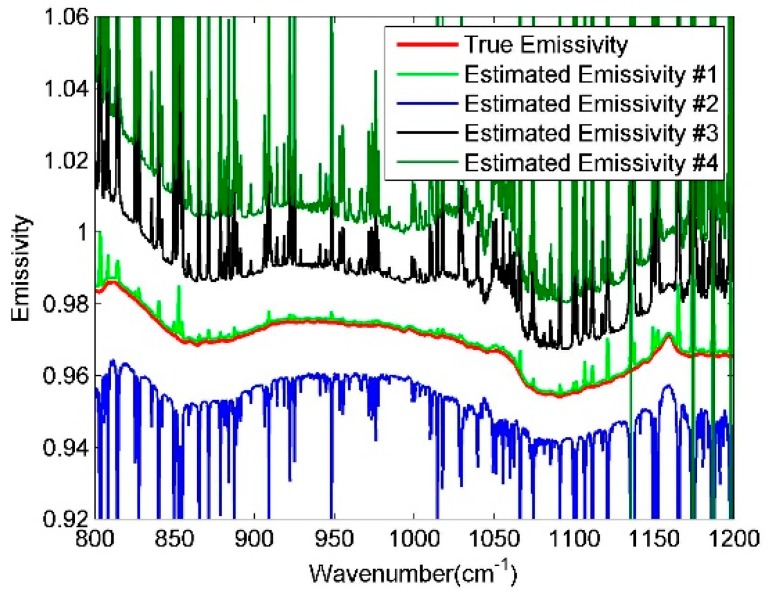
Diagrammatic sketch of soil emissivity estimation. The red dot line is the actual emissivity spectrum, while the other lines are the estimated spectra. Estimated Emissivity #1 represents the estimated spectrum calculated with the max (Tg_λ_) using Equation (4). The true land surface temperature (LST) is varied, with +0.5 k, −0.5 K, and −1 K as the estimated LST value, Estimated Emissivity #2, #3, and #4 represent the corresponding estimated spectra.

A soil emissivity sample (red-orange sandy loam) chosen from the ASTER spectral library is given in Figure 3 (red solid line) together with the corresponding estimated LSE [24]. The true LST is varied between −1 and +0.5 K in steps of 0.5 K as the estimated LST value. The ground-level brightness temperature (Tg) that varies in bands (λ) is defined by B (λ,Tg) = L_sur_ (λ), the maximum Tg value in N bands (max (Tg_λ_)) is also adopted to be the estimated LST and represented in Figure 3. Figure 3 shows that the characteristics of the atmospheric downwelling radiation in LSE estimation spectrum will be lower if the estimation of the surface temperature is more accurate, also max (Tg_λ_) is close to true LST. Finally, max (Tg_λ_) is adopted to be the estimated LST value, the estimated LSE result calculated from Equation (4) is designated as ($\tilde{\mathrm{LSE}}$).

**Figure 4:**
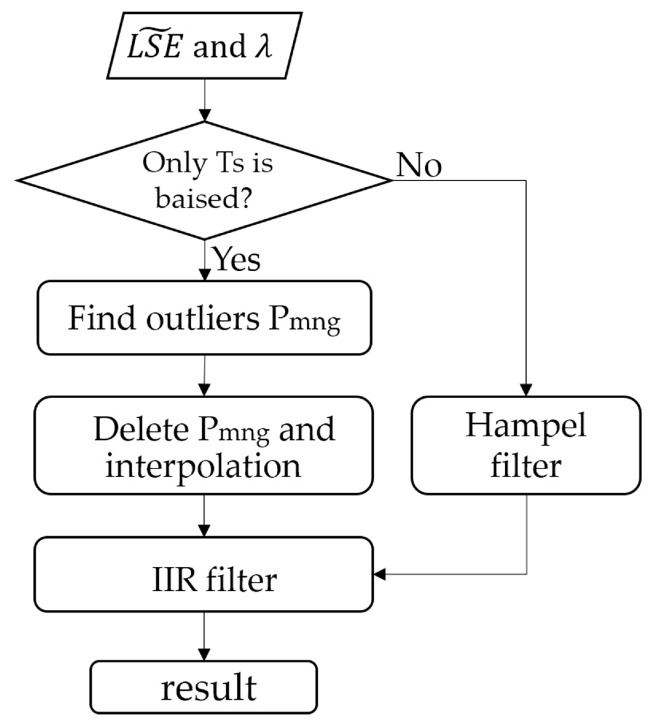
Flow diagram for the pre-estimate shape procedure.

In order to obtain the LSE shape estimation, a pre-estimate shape procedure (Figure 4) is first adopted to restore the shape and determine most of the crest or trough information. 

**Figure 5:**
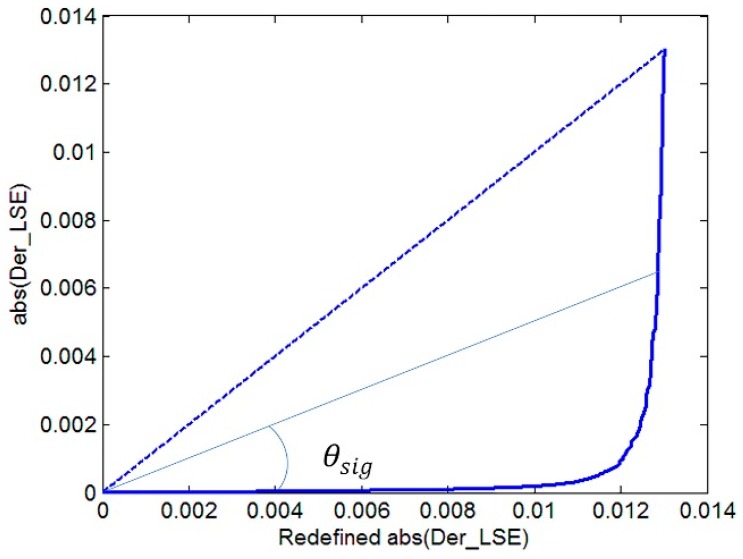
Diagrammatic sketch of Der_LSE.

When max (Tg_λ_) is adopted as the LST estimation in Equation (4), the estimated LSE crest or trough position is basically unchanged, except for the channels of peak portion influenced by atmospheric information. Based on this, the purpose of our program is to remove the peaks and then take a smooth overlay to obtain most of the crest or trough positions. The absolute value of first-order difference (abs(Der_LSE)) in $\tilde{\mathrm{LSE}}$ between two adjacent channels is firstly calculated. The outliers can be removed by distinguishing the position where abs(Der_LSE) changes sharply, by setting a threshold. The threshold value cannot be set to a fixed value because of the uncertainty of abs(Der_LSE) with different kinds of LSE or max (Tg_λ_). In this paper, the abs(Der_LSE) is sorted in ascending order as the ordinate, then we record the ordinal number of each sorted value as the abscissa. Afterwards the range of abscissa is readjusted as [min(abs(Der_LSE)) max(abs(Der_LSE))] with a step delta_m = (max(abs(Der_LSE)) – min(abs(Der_LSE)))/length(abs(Der_LSE)), where length(abs(Der_LS E)) is the number of abs(Der_LSE). When the origin is connected to each point (abs(Der_LSE)) on the difference curve, every obtained straight line has an included angle ($\theta_{\mathrm{sig}}$) with the abscissa. This definition makes all the abs(Der_LSE) data lines fall within the 45-degree included angle (dotted line in Figure 5 with the horizontal axis), which is uniformly defined for comparison. If $\theta_{\mathrm{sig}}$ is larger than the mean value of $\theta_{\mathrm{sig}}$ multiplied by A (a constant), the corresponding Der_LSE value set is defined as P_m_, also recording the channel position i. According to a large amount of data testing, A is set to 0.414.

Using only the first-order difference will cause a misjudgment of the outliers, it will eliminate the peaks and troughs that originally exist in the wider wavelength range of the true LSE. Therefore, the absolute value of second-order difference between the elements of $\tilde{\mathrm{LSE}}$ is used. The processing flow is the same as that of first-order difference method previously mentioned above but allows determining of a second outliers set, to be referred to here as P_n_. After two treatments to obtain the outliers, we take the intersection of the two types of outliers, resulting in a set to be called P_mn_. At the same time, when the number of channels between each two adjacent positions (P_mn_${}_{{|}_{i}}$ and P_mn_${}_{{|}_{i+a}}$, where a is a constant) is less than five channels (a < 5), all channels between two adjacent positions will be considered as outliers, to be referred to as P_g_. Finally, the outlier is the union of P_g_ and P_mn_. Afterwards, the trajectory of the curve is obtained via spherical linear interpolation after the outliers are removed. The interpolated data have a small amount of noise and are designed to be processed by a 12-stage infinite impulse response (IIR) zero-phase delay low-pass filter [25]. 

**Figure 6:**
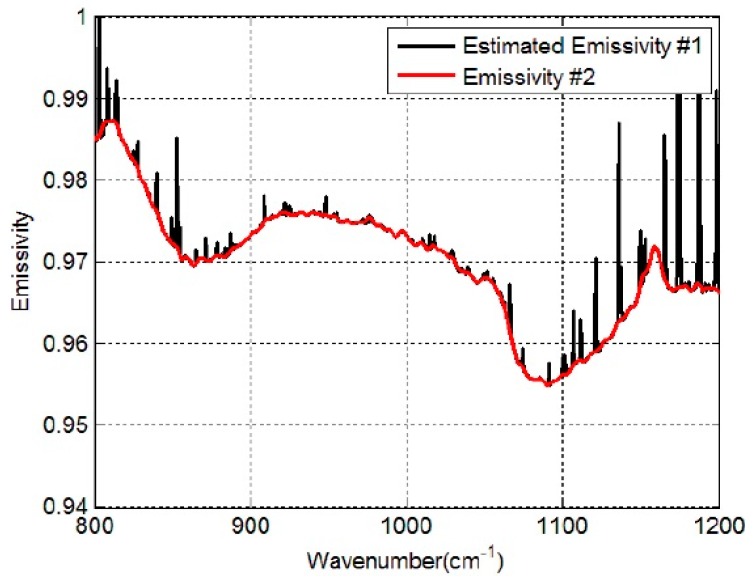
Estimated Emissivity #1 represents the estimated LSE calculated with the max (Tg_λ_) using Equation (4) (Black line). Red line (Emissivity #2) is the estimated shape of LSE using the pre-estimate shape procedure.

When the data contain at-ground radiance error (random noise) and downwelling radiance error, the estimated emissivity loses some shape information. In that case, the Hampel filter is adopted. The Hampel filter block detects and removes the outliers of the input signal by using the Hampel identifier [26]. The Hampel identifier is a variation of the three-sigma rule of statistics, which is robust against outliers. For each sample of the input signal, the block computes the median of a window composed of the current sample and (Len−1)/2 adjacent samples on each side of the current sample (Len is the window length you specify through the window length parameter). After the correction of the IIR zero-phase delay low-pass filter, the Hampel filter further smooths the curve. Figure 6 shows the result of the pre-estimate shape algorithm of the soil spectrum mentioned in Figure 2. When the pre-estimate shape algorithm is finished, we can get a new segmentation scheme based on the recovered LSE shape and further use the LSEC algorithm to retrieve LST and LSE, also we henceforth refer to this pre-estimate shape of the LSEC algorithm as the PES-LSEC method.

## 3. Experiments

The PES-LSEC method is tested with simulated data and in situ data. The simulated data are prepared using in a simulation model (4A/OP, operational release for 4A radiative transfer model) with spectral (ASTER library) and climatological libraries to produce the necessary elements in Equation (2). In situ data are used for the evaluation of the PES-LSEC method.

### 3.1. Simulated Data

To simulate the hyperspectral thermal infrared variables in Equation (2), the 4A/OP model is adopted to calculate the transmittance, upwelling radiance, and downwelling radiance using input atmospheric profiles and the emissivity database. The 4A model is a line-by-line model which allows fast simulation of radiative transfer, particularly over the infrared range with a “pseudo-infinite” (high) resolution [27]. In this experiment, the spectral range is 800–1200 cm^−1^, with a spectral resolution of about 0.5 cm^−1^ and sampling interval of 0.25 cm^−1^. The spectral response function is the same as that of the IASI sensor.

**Figure 7:**
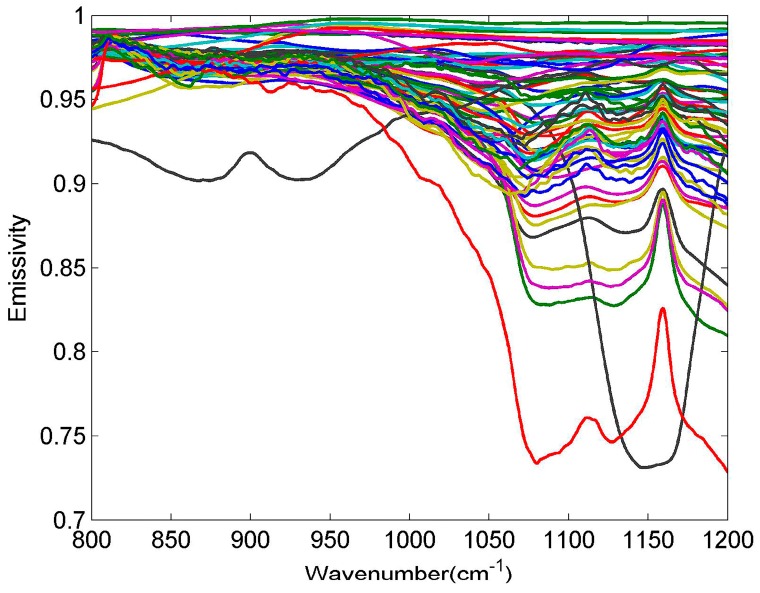
Selected emissivity spectra from the ASTER spectral library.

The input atmospheric profiles are obtained from the Thermodynamic Initial Guess Retrieval (TIGR) data set, which includes a climatological library of 2311 representative atmospheric situations classified into five airmass types (Tropical, temperate -Midlat1-, cold temperate and summer polar-Midlat2-, Northern Hemisphere very cold polar -polar1-, and winter Polar -Polar2-) [28]. The selected 946 clear sky atmospheric profiles are used for simulation experiments, while the bottom atmospheric temperature of the profiles is adopted as LST. Meanwhile, 65 spectra (52 soil types, four vegetation types, nine water/snow/ice types) from the ASTER spectral library are collected to describe most of the features appearing in the terrestrial ecosystem (Figure 7).

For LST and LSE retrieval results, the temperature bias and root-mean-square error are adopted to characterize the method accuracy:

ΔT_s_ = T_ret_ − T_true_, (5)

where ΔT_s_ is the difference of the retrieved temperature (T_ret_) and the actual temperature (T_true_). RMSE_T_ is the root-mean-square error of the retrieved LST and actual LST, while N_D_ is the number of total samples.

(6) $${\mathrm{RMSE}}_{T}\;=\sqrt{\frac{\sum_{i=1}^{N_{D}}{{(T}_{\mathrm{ret},i}{-T}_{\mathrm{true},i})}^{2}}{N_{D}}},$$

(7) $${\mathrm{RMSE}}_{\varepsilon ,j\;}=\sqrt{\frac{\sum_{i=1}^{N_{D}}{{(\varepsilon }_{\mathrm{ret},i,j}{-\varepsilon }_{\mathrm{true},i,j})}^{2}}{N_{D}}},$$

(8) $${\mathrm{RMSE}}_{\varepsilon \;}=\sqrt{\frac{\sum_{i=1}^{N_{D}}\sum_{j=1}^{N_{M}}{{(\varepsilon }_{\mathrm{ret},i,j}{-\varepsilon }_{\mathrm{true},i,j})}^{2}}{N_{D}\cdot N_{M}}}.$$

RMSE_ε,j_ is used to evaluate the algorithm emissivity retrieval accuracy (rmse) in each band, where ε_ret_ is the retrieved emissivity, ε_true_ is the true emissivity. N_M_ is the number of bands, the rmse of the retrieved and actual emissivity difference (RMSE_ε_) can be described using Equation (8).

**Figure 8:**
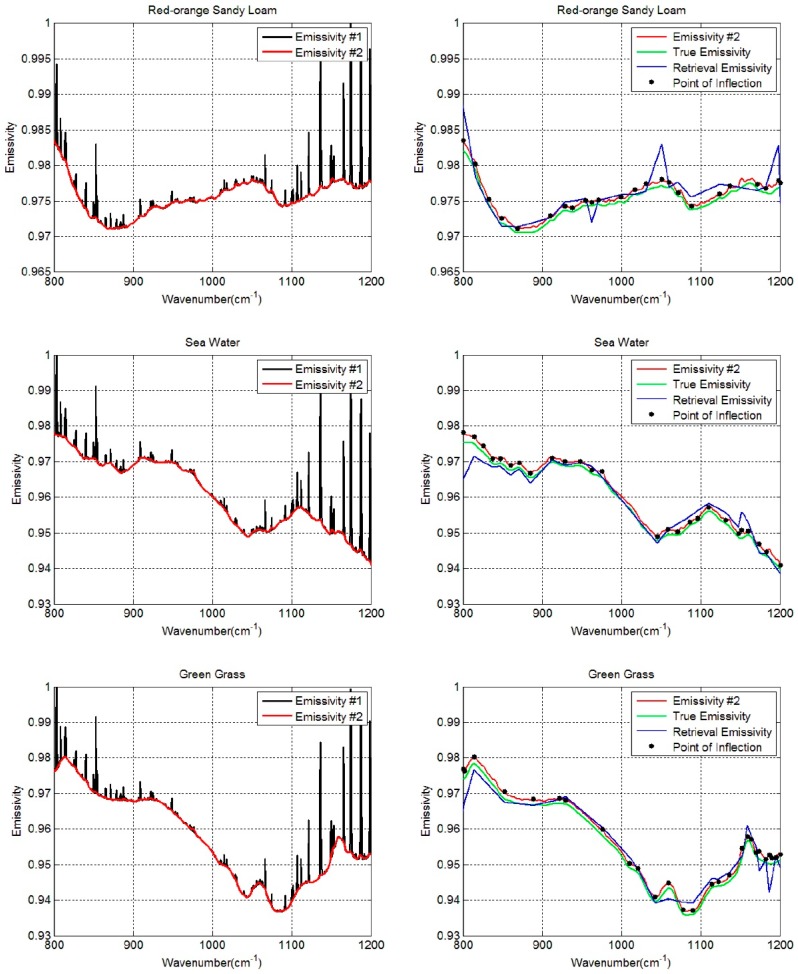
Retrieval results of three spectra (red-orange sandy loam, sea water, and green grass) using PES-LSEC method. The calculation results of Equation (4) with max (Tg_λ_) are drawn in black lines (Emissivity #1). Red lines (Emissivity #2) are the estimated shape of LSE using the pre-estimate shape procedure. Black points are the identified inflection points. Green lines are the true emissivity values used in the simulation. Blue lines are the final retrieval results of emissivity using PES-LSEC method.

The prepared data are first processed using the pre-estimate shape procedure under the condition that only LST was biased. Figure 8 shows the initial shape estimate results of three types of land surface (red-orange sandy loam, sea water, and green grass): the reconstructed data (Emissivity #2, red lines) have a similar tendency to that of the true LSE (green lines). Furthermore, most of the crest and trough positions of true LSE are preserved, which is crucial for subsequent segmentation processing. Secondly, the first and second derivatives are used to identify the inflection points to provide new segmentation schemes for the aforementioned three spectra demonstrated in Figure 8 (black points). Afterward, the non-equal-interval LSE segmentation scheme was integrated into the LSEC algorithm (PES-LSEC method) to obtain the retrieval emissivity shown in Figure 8 (blue lines). As expected, the new segmented scheme according to the position of inflection points better fits the true LSE curves and obtains satisfactory results. These examples present the condition that max (Tg_λ_) is close to true LST using one typical atmospheric profile, so the Emissivity #2 (red lines) shows a good agreement with the true emissivity. When the deviation between max (Tg_λ_) and Ts is large, the identified red line does not exactly match the true curve shape; however, it can keep most of the inflection point information that provides satisfactory emissivity result after using the PES-LSEC method. Also, we can find the crest and trough positions are larger than the real values for some emissivity spectra, for example the green grass in the 1150–1200 cm^−1^ region. This is a normal phenomenon, as some small outliers are retained without reaching the set threshold. This will cause a slight the increase in the number of LSE segments and in the computation time. Nevertheless, the retrieval accuracies of LST and LSE will be improved to some extent in the linear emissivity spectra fitting process. 

**Figure 9:**
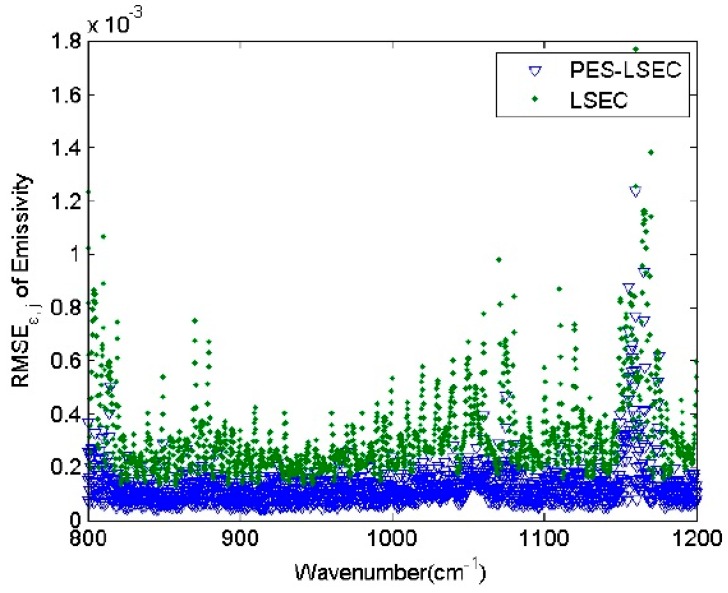
RMSE_ε__,j_ of the two methods.

Table 1 represents the retrieval accuracy of LSEC and PES-LSEC method for three land surface types (red-orange sandy loam, sea water, and green grass) corresponding to Figure 8. The rmse of the retrieved emissivity and LST with the PES-LSEC method are better than LSEC method, but slightly. When all 946 atmospheric profile types and 65 emissivity spectra for the PES-LSEC method are considered, the RMSE_T_ is 0.001, which is smaller than the LSEC retrieval accuracy (0.005). ΔT_s_ is within [−6.0 × 10^−3^, 6.0 × 10^−3^] K. The root-mean-square error in each channel (RMSE_ε,j_) is shown in Figure 9 for the two methods LSEC and PES-LSCE. In the 800–1200 cm^−1^ region, with the PES-LSCE method, RMSE_ε,j_ is smaller than 0.0014, and smaller than for the LSEC method (0.0012). When the errors of at-ground radiance and downwelling radiance are not considered, our method has a better performance on the shape estimation, providing a reasonable segmentation scheme and a smaller retrieval error.

**Figure 10:**
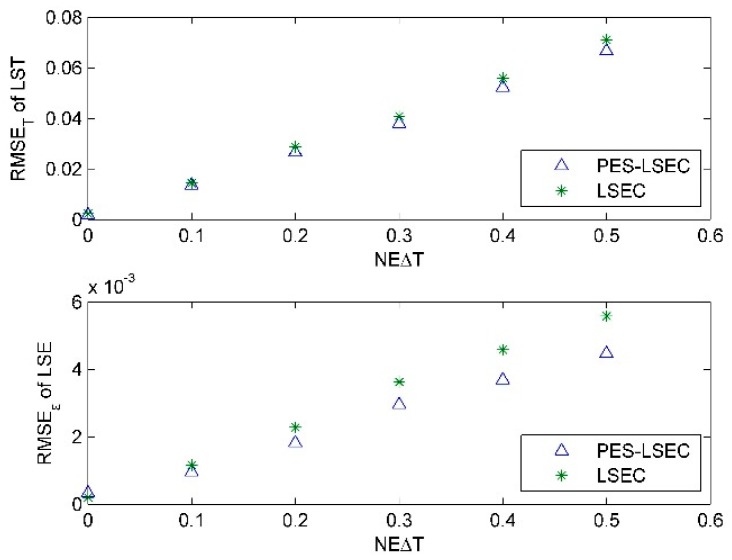
RMSE_ε_ and RMSE_T_ for LSEC and PES-LSEC method with the at-ground radiance error.

The required inputs for the algorithm are the ground-leaving radiance and atmospheric downward radiance, which are available after the atmospheric correction process. Atmospheric correction is a key issue, and there are always errors associated with it. The errors related to these two quantities can propagate into the derived surface temperature and emissivity. The potential errors include random instrumental noise, instrument calibration error, and atmospheric downward radiance error. In this paper, because we deal mainly with the at-ground level, only the at-ground radiance error and downwelling radiance error are discussed. Different levels of noise NEΔT (NEΔT = 0.0, 0.1, 0.2, 0.3, 0.4, and 0.5 K) were added to the simulated at-ground radiance. As shown in Figure 10, the PES-LSEC method does not greatly improve the retrieval accuracy of LST nor of LSE, with NEΔT = 0.0, 0.1, 0.2, compared to that of LSEC. Whereas, when NEΔT increases to 0.5 K, RMSE_ε_ with the PES-LSEC method is 0.0045, and RMSE_T_ is 0.07. The PES-LSEC method has a similar performance to that of the LSEC method for the retrieval error on LST but improves the accuracy of the LSE error. When the at-ground spectral radiance error is added to the simulated data, the random error results in partial shifts of the crests and troughs, leading to a loss of most information. Our pre-estimate shape procedure can reconstruct the basic trend of the curve, which is similar to that of the actual emissivity and is relatively smooth. This smoothed curve leads to a significant reduction in the number of segments but a large error increase in LSE and LST. However, when the number of segments is increased to be similar to the number of segments of the LSEC scheme, the performance is nearly the same in terms of the LSEC accuracy.

**Figure 11:**
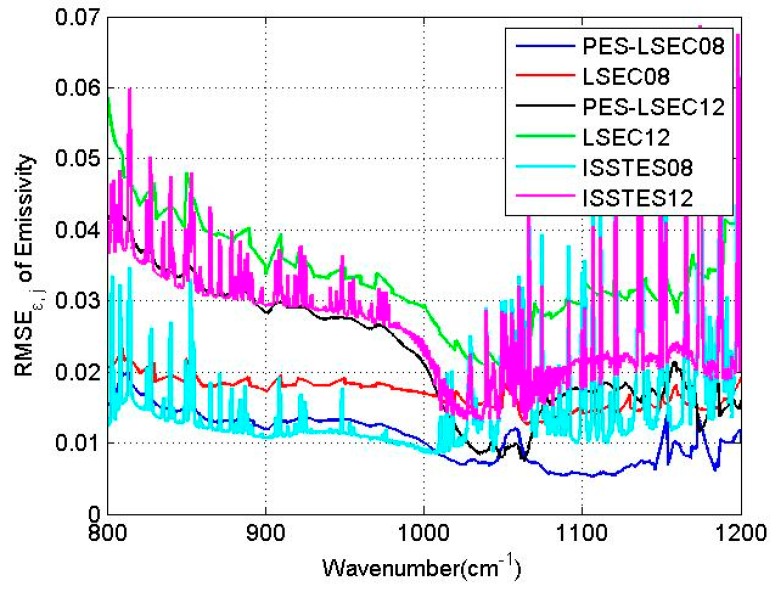
RMSE_ε,j_ for the 800–1200 cm^−1^ region. The blue and black lines are the retrieval results of the PES-LSEC method with the scale factors of moisture profile being −0.2 and 0.2, respectively. The red and green lines are the LSEC results with the scale factors of moisture profile −0.2 and 0.2, respectively. ISSTES08 and ISSTE12 present the RMSE_ε,j_ of emissivity using ISSTES method with the scale factors of moisture profile −0.2 and 0.2, respectively.

To investigate the influence of the atmospheric downward radiance error on the accuracies of land surface temperature and emissivity retrieval, the moisture profiles from the TIGR database were multiplied by 80% and 120%, leaving the shape of the humidity profile unchanged, but generating the simulated downwelling radiance with error. Figure 9 shows the retrieval accuracy of LSE in each band when the moisture profiles is not biased. When the moisture profiles are shifted by 0.8 and 1.2, RMSE_T_ are 1.11 K and 1.14 K, respectively, using the PES-LSEC method. When only the LSEC method is used, RMSE_T_ are 1.2 and 1.4 K, respectively. RMSE_ε,j_ of the PES-LSEC method in each band is mostly below 0.02 when the moisture profiles are shifted by −20% and is mostly less than 0.04 when the moisture profiles are shifted by +20% (Figure 11), better than the LSEC retrieval results. Compared with the ISSTES method, LSEC and PES-LSEC show good noise-resistant ability especially in 1050–1200 cm^−1^ region. However, with the influence of the moisture profile error, the atmospheric downward radiance error still has a bigger effect on LST and LSE retrieval with the PES-LSEC method. 

### 3.2. In Situ Data

**Figure 12:**
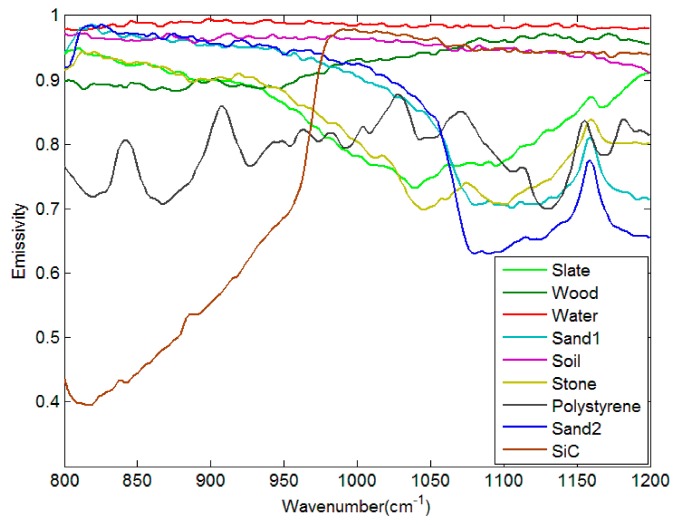
Emissivity spectra of nine samples.

In order to validate the proposed separation method of LST and LSE, the in situ data were collected from PIRRENE (Program Interdisciplinaire de Recherche sur la Radiométrie en Environnement Extérieur), site of ONERA (Office National d’Etudes et de Recherches Aérospatiales) center of Fauga-Mauzac [29]. These data are nine samples of field thermal infrared spectra measurement data, which are slate (homogeneous and flat piece of slate), wood (plywood), water, sand1 (Morocco sand), soil (soil from Negev desert), stone (flat rough and homogeneous rock), pstyr (Extruded polystyrene), sand2 (Fontainebleau type sand), and SiC (SiC powder. Grain size ~120 μm). Figure 12 gives the average emissivities of the nine samples that were measured at different times (3:00, 4:00, 7:00, 8:00, 9:00, 10:00, 13:00, 15:00, and 16:00) for two days. A detailed description of the ground test can be found in [29,30].

In the field-measured experiment, the data obtained via direct measurement include: (1) nine samples and labsphere infragold plate leaving-surface spectral radiances measured by the BOMEM (MR250 Serie) instrument (the spectral resolution was 4 cm^−1^, and the sampling interval was 2 cm^−1^); (2) nine sample temperatures measured by the broadband long-wave infrared radiometer (KT19); (3) infragold plate temperature measured by the thermocouple; and (4) reflectance of infragold plate and nine sample emissivities measured in laboratory. Directly measured data needs to be initially processed to obtain the downwelling radiation. Meanwhile, in order to be able to compare them with the actual temperature of the sample, the true value of the sample temperature must also be calculated from KT19 measurements. Therefore, preprocessing of measured data is essential; the detailed process can be found in [29,30].

As the resolution is different from the simulated data, the parameter A in the pre-estimate shape procedure is reset to 2. To validate the accuracy of the retrieval LSE, an index Mean_εt_, is used to analyze the emissivity retrieval accuracy of algorithm; the equation is:

(9) $${\mathrm{Mean}}_{\varepsilon t\;}=\;\frac{\sum_{i=1}^{N_{D}}\sum_{j=1}^{N_{M}}{|\varepsilon }_{\mathrm{ret},i,j}{-\varepsilon }_{\mathrm{true},i,j}|}{N_{D}\cdot N_{M}},$$

where ε_ret,i,j_ is the retrieved emissivity of the ith sample at wavenumber j, ε_true,i,j_ is the actual emissivity of the ith sample at wavenumber j.

**Figure 13:**
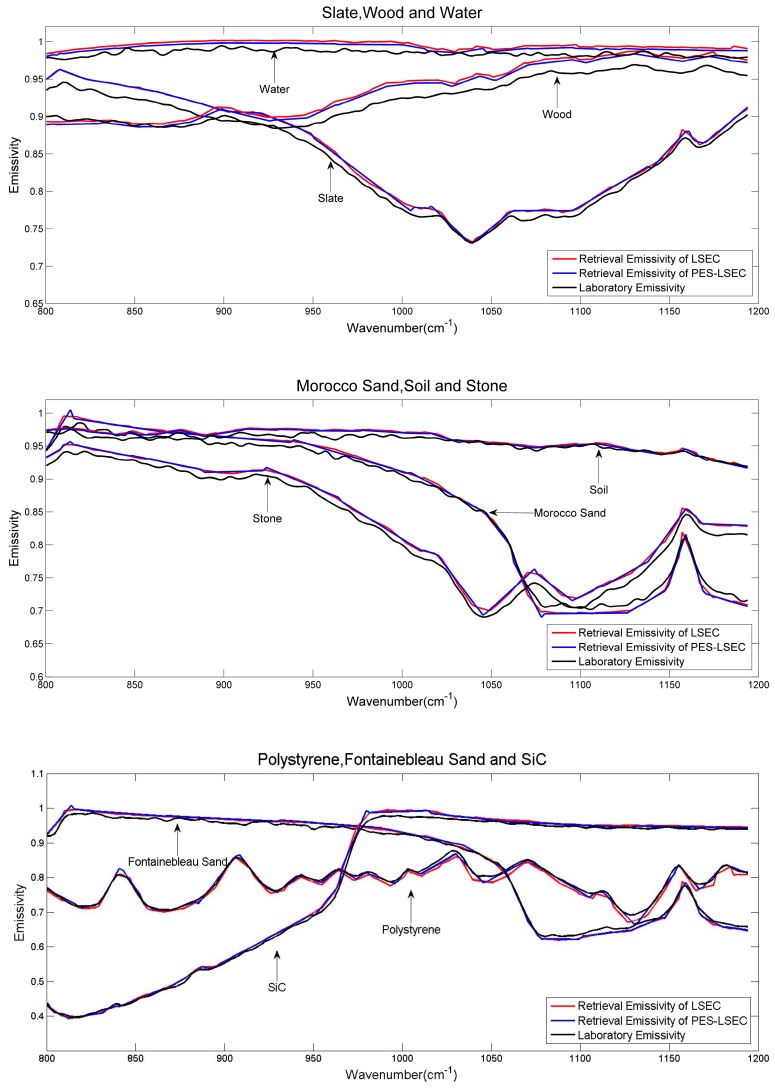
Laboratory emissivities of the nine samples and retrieved emissivities using the LSEC and PES-LSEC method.

**Figure 14:**
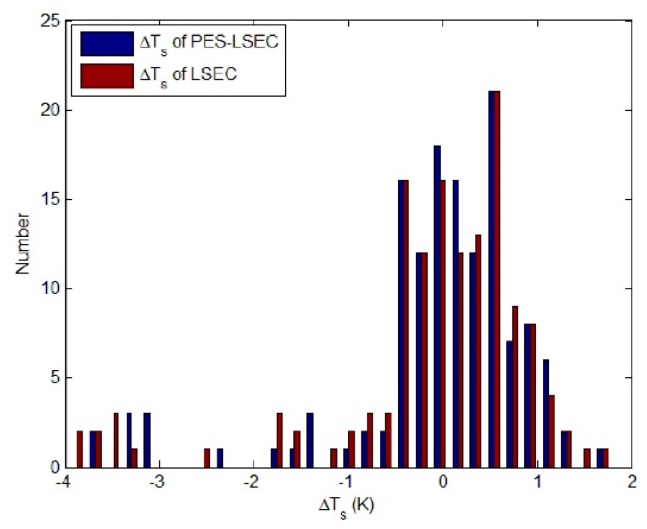
ΔT_s_ of nine samples.

**Figure 15:**
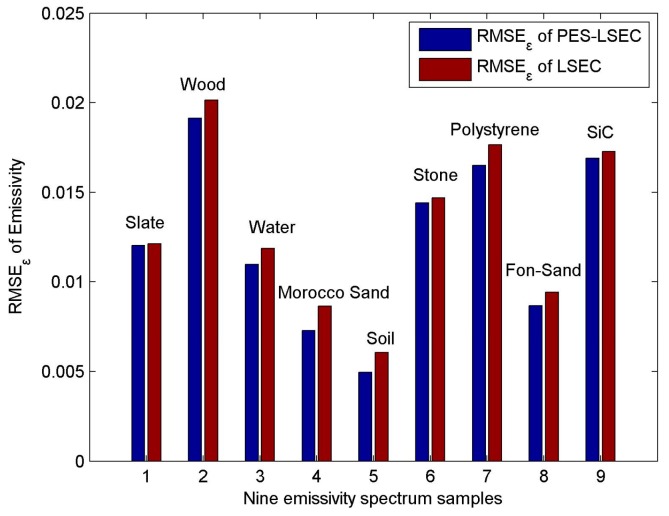
RMSE_ε_ of nine emissivity spectrum.

RMSE_T_ of the PES-LSEC method is 0.9 K better than the LSEC’s accuracy (1.1 K). Mean_εt_ of the PES-LSEC method and LSEC method are 0.01 and 0.012, respectively. Retrieval results of the nine sample emissivity spectra using the LSEC and PES-LSEC method are shown in Figure 13. To clearly demonstrate the performances of the LSEC and PES-LSEC methods, ΔT_s_ of two separation algorithms and RMSE_ε_ for each sample are drawn on Figure 14 and Figure 15, respectively. The inversion value and the measured value for LSE are found to be in good agreement to a large extent, and LSE values of most samples are slightly overestimated. Also, the PES-LSEC method’s errors with the nine samples follows the LSEC retrieval emissivity to a certain degree. The PES-LSEC method shows good performance in identifying the inflection point, for the polystyrene sample, PES-LSEC better preserves the crest and trough position around 1050 cm^−1^, 1113 cm^−1^, 1170 cm^−1^, and 1180 cm^−1^ channel than the LSEC method, thus improving the accuracy to some extent. Soil, wood, Morocco sand, and water also present a certain degree of improvement. In addition, the PES-LSEC method achieves similar accuracy with the LSEC using large segmentation interval. The sample’s spectrum of slate, stone and SiC are relatively smooth, so a large segment is identified. For example, in the 920–1000 cm^−1^ interval, the slate and stone spectra are only divided into two segments, SiC is only divided into three segments in the range of 1020–1190 cm^−1^. For these three samples in Figure 15, we can find the accuracy of the PES-LSEC and LSEC methods are basically the same. This is mainly because the PES-LSEC method uses a segmentation scheme with large segments, and some small peak-to-valley information is ignored, resulting in less precision improvement. As suggested in the LSEC method, five channels in one segment can better fit the LSE even if some peak points are lost. 

There are still some other problems when compares the retrieval results with the laboratory emissivity. For example, the uncertainty of the sample emissivity laboratory measurement may cause a deviation from the land surface temperature calculated via KT19. Meanwhile, the instantaneous fields of view (IFOV) of BOMEM and KT19 are not the same, resulting in different observation sample areas. The heterogeneity and non-isothermality of the sample itself also cause differences in the temperature observations. Generally, the PES-LSEC method has a good performance with the in situ data, it keeps most of the crest and tough information of emissivity. The LST retrieval accuracy of most samples is within 1 K. Although, wood, polystyrene, and SiC presents a bigger RMSE_ε_ than other samples, and the retrieval emissivity are basically consistent with the tendency of laboratory LSE. 

## 4. Conclusions

A temperature and emissivity separation method (PES-LSEC method) was proposed based on the linear constraint of LSE to reduce the number of unknowns, making the underdetermined equation solvable with accurate atmospheric correction. In this paper, a pre-estimate shape method was first adopted to provide the basic tendency of LSE and determine relatively accurate crest and trough positions, producing a better non-equal-interval segmentation scheme. The PES-LSEC method is then used to separate the LST and LSE.

The numerical experiments are first used to investigate the accuracy of the algorithm and to carry out sensitivity analyses. A total of 946 atmospheric profiles from the TIGR database and 65 natural surface materials from the ASTER spectral library are combined together to generate the simulated data set. When noise of NEΔT = 0.5 K is added to the at-ground radiance of simulated data, RMSE_ε_ and RMSE_T_ are 0.0045 and 0.07, respectively. When the moisture profiles are shifted by ±0.2, RMSE_T_ are 1.11 and 1.14 K, respectively. RMSE_ε,j_ of the retrieved emissivities of the PES-LSEC method are mostly below 0.02 and 0.04 in each channel, which is better than the LSEC retrieval results.

The results of the sensitivity analysis show that at-ground radiance error equivalent of 0.5 K has a significant influence on emissivity retrieval and a relatively small influence on surface temperature retrieval, in comparison with those for the LSEC method. The atmospheric downward radiance error has big impacts on temperature and emissivity estimation, but our algorithm can achieve better retrieval accuracy of temperature and emissivity than those of the LSEC method.

The PES-LSEC algorithm is also used to retrieve surface temperature and emissivity with in situ measurements. With initial-shape estimation, more accurate and less segmentation schemes were given to obtain a better accuracy than that of the LSEC method. The crest and tough information of LSE is better preserved using PES-LSEC method. As for the LST and LSE retrieval accuracies, they have been improved to some extent. Most of samples of the errors of LST are within 1 K, and the root-mean-square-error of LSE of every sample has been found to be less than 0.02.

Finally, even though we assume that the atmospheric effect of the remotely sensed data has been successfully corrected, our method fits only for the at-ground level, and a new step will be to reach the satellite level. The unknown errors of upwelling radiance and transmittance of the atmosphere will make the pre-estimate shape procedure invalid and unable to determine the basic tendency of emissivity. Therefore, studies are still to be done in the future, for example, on how to determine the shape of the LSE at the satellite level to separate LST and LSE. Tests with neural network technology show that the constructed network strongly relies on the sample spectral library of emissivity. The more comprehensive the land surface type information covered by the spectral library, the more accurate the estimated LSE. Moreover, accurate atmospheric correction is necessary in our method, therefore how to correct for the atmospheric effects accurately is another issue to improve the LST retrieval accuracy.

## Figures and Tables

**Table 1:** Retrieval accuracies of LSE and LST (RMSE_ε_ and RMSE_T_) using LSEC and PES-LSEC method.

	LSEC Method		PES-LSEC Method	
	RMSE_ε_	RMSE_T_ (T)	RMSE_ε_	RMSE_T_ (T)
Red-orange sandy	6.2 × 10^−5^	5.5 × 10^−4^	4.4 × 10^−5^	3.1 × 10^−4^
Sea water	1.9 × 10^−4^	1.0 × 10^−3^	8.7 × 10^−5^	5.2 × 10^−4^
Green grass	2.1 × 10^−4^	8.1 ×10^−4^	1.2 × 10^−4^	3.8 × 10^−4^
